# Effects of terrigenous sediment on settlement and survival of the reef coral *Pocillopora damicornis*

**DOI:** 10.7717/peerj.387

**Published:** 2014-05-15

**Authors:** Kaipo Perez, Kuʻulei S. Rodgers, Paul L. Jokiel, Claire V. Lager, Daniel J. Lager

**Affiliations:** Hawaiʻi Institute of Marine Biology, Kāne’ohe, HI, USA

**Keywords:** Sediment, Coral, Larvae, Settlement, Reproduction, Survival

## Abstract

Survival and settlement of *Pocillopora damicornis* larvae on hard surfaces covered with fine (<63 µm) terrigenous red clay was measured in laboratory Petri dishes. The dishes were prepared with sediment films of various thicknesses covering the bottoms. Coral larvae were incubated in the dishes for two weeks and the percent that settled on the bottom was determined. There was a statistically significant relationship between the amount of sediment and coral recruitment on the bottom, with no recruitment on surfaces having a sediment cover above 0.9 mg cm^−2^. Experimental conditions for the delicate coral larvae were favorable in these experiments. Total survival over the two week settlement tests expressed as the sum of coral recruits and live larvae at the end of the experiment did not show a significant decline, so the major impact of the sediment was on successful settlement rather than on mortality. Larval substrate selection behavior was the primary factor in the observed result.

## Introduction

Sedimentation has been identified as a major detrimental factor on coral reefs (Johannes, 1975; Cortes & Risk, 1985; Rogers, 1990; Grigg & Birkeland, 1997). Fabricius (2005) noted that evaluation of the impact of terrestrial runoff on coral reef ecology is very difficult even though sedimentation has a very high impact relative to other processes. Extreme sedimentation can smother and kill corals (Edmondson, 1928). Suspended sediment reduces irradiance, restricts photosynthesis and negatively affects coral growth (Davies, 1991; Anthony & Connolly, 2004). An inverse relationship has been established between sediment loading and coral larval survival and development (Gilmour, 1999). Research has also identified lethal and sub-lethal effects to corals from substances associated with sediments such as pesticides, fertilizers, and petroleum products (Glynn et al., 1989; Te, 2001). Particles can act as substrate for these chemicals and other contaminants. These pollutants carried in sediments can affect settlement, recruitment, and survivorship of coral and their larvae. Even low levels of these toxins have been shown to dramatically affect morphology and physiological processes of corals (Glynn et al., 1986).

Observations indicate that coral planulae do not settle on silt covered surfaces (Harrigan, 1972). Rogers (1990) summarized the existing literature and concluded that ‘normal’ sedimentation rates for coral reefs appear to be on the order of 10 mg cm^−2^ d^−1^ or less, with typical suspended solids concentrations less than 10 mg l^−1^. She identified the need to determine the quantity of different types of sediment that will deter coral settlement. Lewis (1974) reported that a sprinkling of fine washed sand in the bottom of test bowls reduced settlement to less than one third of the settlement rate on a clean glass surface. Hodgson (1991) studied coral settlement on a glass surface that was only partially covered with a mixture of sand, silt and clay. In this situation he found that the larvae will still settle on areas that are clear of sediment. Te (1992) cultured larvae of the coral *Pocillopora damicornis* to four concentrations of sediment (0, 10, 100, 1000 mg l^−1^) for 14 days under two contrasting water agitation levels and found no significant difference in larval settlement on the glass walls of the containers. Presumably the vertical glass walls did not accumulate sediment, so there was no difference in substrate between the treatments. Babcock & Davies (1991) used sediment composed of fine sand and silt of mixed terrigenous and carbonate origin to determine the effect of various rates of sedimentation (0.5–325 mg cm^−2^ d^−1^) on settlement rates of *Acropora millepora* in aquaria. Total number of settled larvae was not significantly affected by sedimentary regime, but higher sedimentation rates reduced coral settlement on horizontal surfaces where sediments could accumulate. More data are needed on the effect of sediment thickness on recruitment of corals onto a surface. As pointed out by Fabricius (2005), the mechanisms by which sediment limits coral development are complex and have been difficult to isolate and quantify. It is clear that coral larvae can survive in highly turbid water and can settle in some situations under conditions of high sedimentation rate. Questions remain as to the quantity of sediment on a surface that will prevent settlement and survival of larvae.

**Figure 1:**
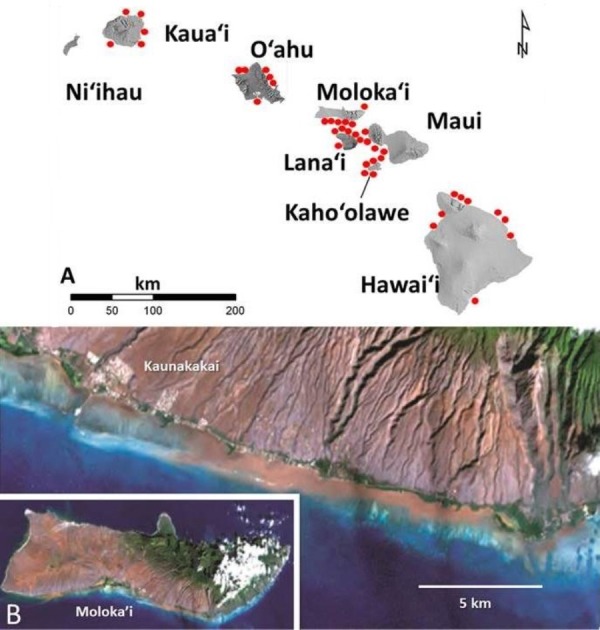
Images of red terrigenous sediment in the Hawaiian islands. (A) Map of the main Hawaiian Islands showing areas with heavy red mud deposits. (B) The Landsat satellite photo taken off south Molokaʻi Hawaiʻi on November 29, 2001 two days after a heavy rainfall showing turbidity plume caused by red muds deposited on the reefs (e.g., Field et al., 2008b).

**Figure 2:**
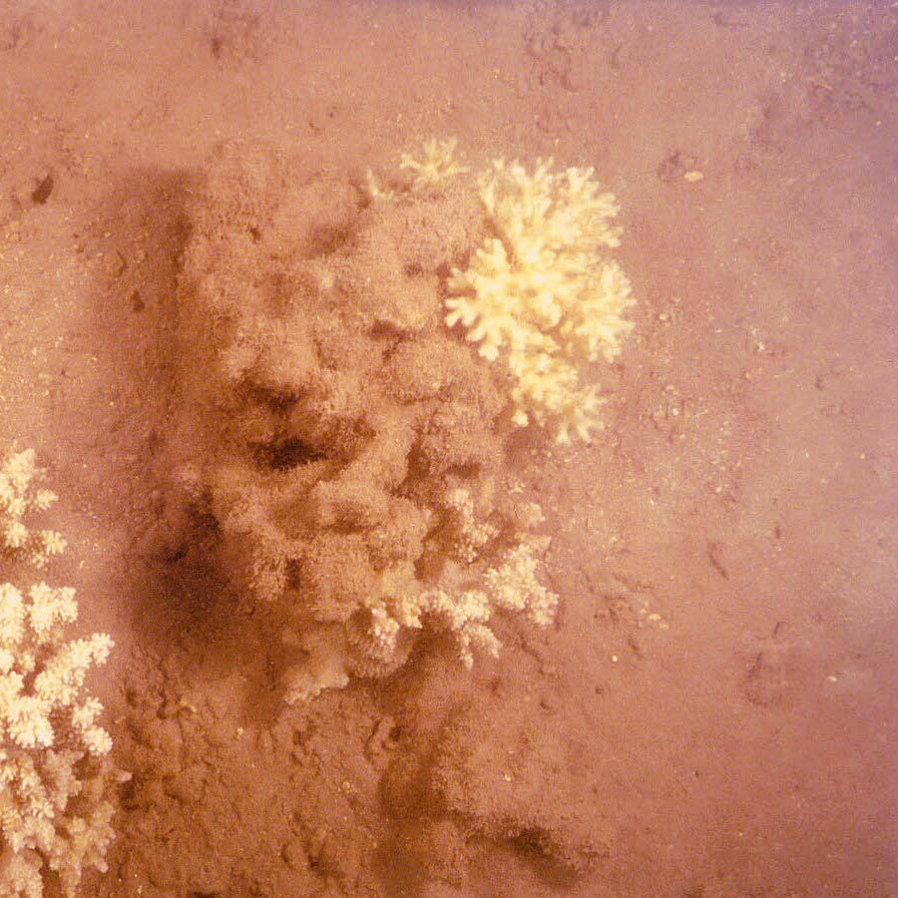
The coral *Pocillopora damicornis* surrounded by hard substrate covered with a film of red mud on turf algae at Molokaʻi, Hawaiʻi.

In Hawaiʻi as in other high islands of the Pacific, flood events transport large amounts of red terrigenous soils onto reefs (Field et al., 2008a). The larger particles settle out quickly but the finest fraction (<63 µm) disperses throughout the reef system (Fig. 1). These fine red muds adhere to surfaces (Fig. 2) as well as form deposits that are continually remobilized by wave events. There are no data on how these fine coatings influence coral settlement and survival of recruits. The purpose of this study was to establish this value using uniform coatings of fine terrigenous red clay muds on hard substrate.

## Materials and Methods

Experiments were conducted in Petri dishes in the laboratory at the Hawaiʻi Institute of Marine Biology located on Moku o Lo‘e Island in Kāne‘ohe Bay, O‘ahu. Settlement of planulae larvae of the reef coral *Pocillopora damicornis* on hard surfaces covered with various amounts of fine sediment was measured.

To avoid chemicals and contaminants associated with soil, soil was collected from a steep undisturbed eroding slope in a remote area near the top of Moku o Lo‘e. This area has never been planted or used for agriculture and is located at a higher elevation than possible sources of anthropogenic contamination. The red subsoil here is typical of the volcanic soils found throughout the high islands of the tropical Pacific region. The soil was thoroughly suspended in seawater using a commercial grout mixer, and screened through a 63 µm sieve. The resulting slurry of fine silt was allowed to settle and the supernatant water siphoned off to produce a dense mud consisting of very fine particles. The stock mud slurry was used throughout the experiments. The wet mud was used to prepare each series of dilutions because drying the mud results in accretions that do not go back into suspension easily. An estimate of the sediment film thickness in the various treatments was obtained. First, the sediment concentration (mg of dry sediment per cm^−3^ of water-sediment film) was measured. A slurry was made from the bulk sediment stirred in a beaker of sea water. The sediment was allowed to settle and the overlying water decanted. Aliquots of 1 cc were taken from the settled sediment layer using a 1 cc syringe and placed in pre-weighed dishes, dried and re-weighed. The sediment thickness in the treatments was calculated as the ratio of weight of dry sediment (g cm^−3^) of the settled wet material to the concentration of dry sediment (g cm^−2^) in the treatment to obtain an estimate of the sediment film thickness (cm). Sediment thickness ranged from 0.008 to 0.08 mm.

*Pocillopora damicornis* is a branching, reef building coral that is commonly found throughout the Pacific and Indian Oceans (Veron, 1986). This species releases abundant positively buoyant planulae on a lunar cycle throughout the year (Jokiel, 1985). Colonies of *P. damicornis* (*n* = 20) were collected from the shallow reef flats surrounding Moku o Lo‘e prior to the full moon and placed in aerated containers. Planulae used in the experiments were collected fresh each morning and transferred to the experimental Petri dishes using a pipette.

**Figure 3:**
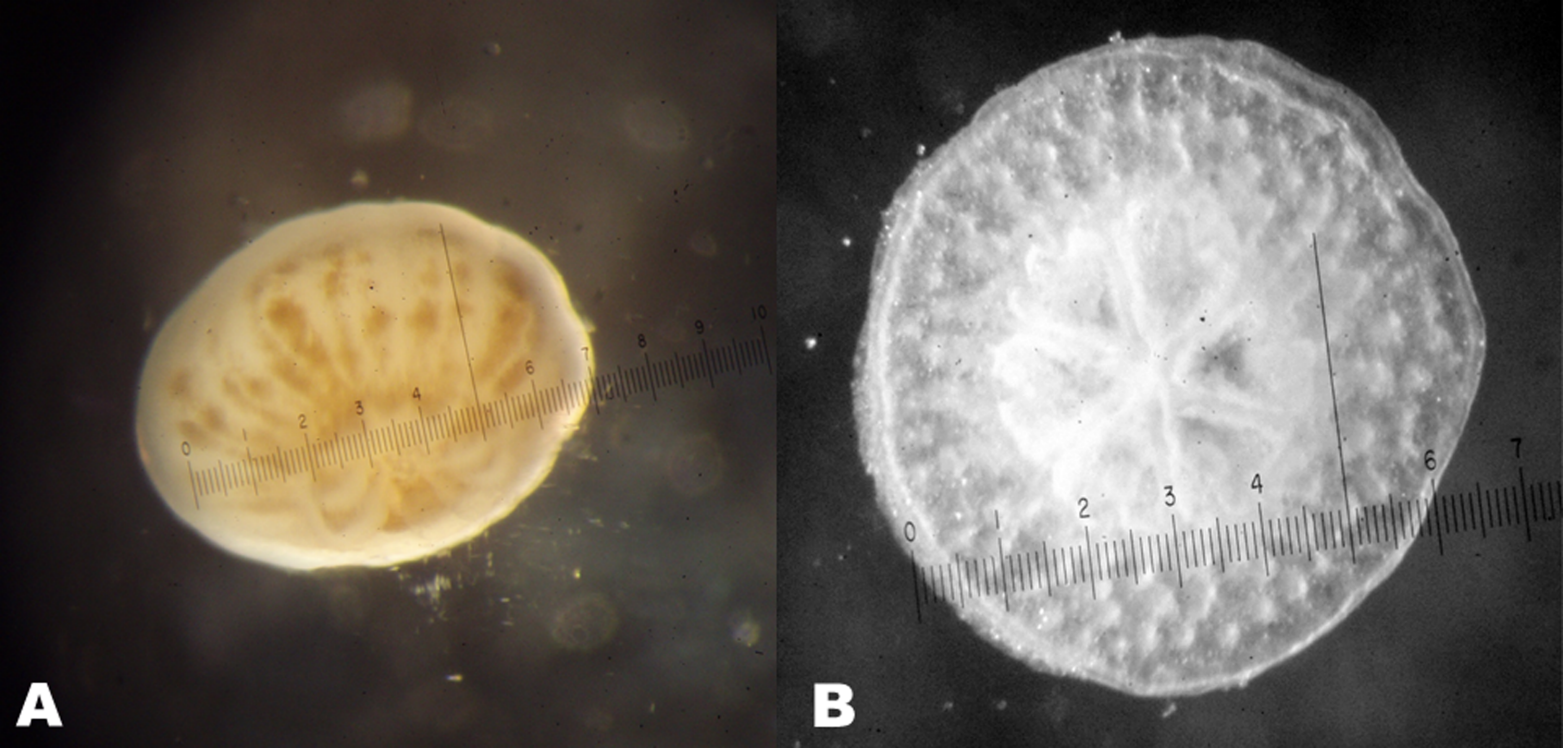
(A) Swimming planula of the coral *P. damicornis.* (B) Settled planula of the coral *P. damicornis*. Scale in µ m.

The experiments were conducted in 100 mm diameter × 15 mm deep Petri dishes that were conditioned in sea water for two weeks prior to introduction of sediment and planulae. Conditioning of the surfaces is necessary because the planulae require a thin biofilm layer for settlement (Richmond, 1985; Baker, 1995; Tran & Hadfield, 2012). A volume of 35 ml of seawater was prepared for each dish in a screw top vial. A treatment with no added sediment served as the control. A sediment concentration series was prepared using 1 drop, 2 drops, 3 drops etc. of stock sediment added to the series of vials. The sediment solutions were homogenized by shaking the vials vigorously before pouring the contents into the pre-conditioned Petri dishes. The sediment was then allowed to settle until the water was clear and a uniform film of silt formed on the bottom over the preexisting biofilm layer. Ten planulae were then gently added to each dish (Fig. 3A). Due to low rates of settlement, the number of larvae was increased to 25 planulae in the subsequent trials. The results were expressed as % of original larvae that settled. Petri dish covers prevented evaporation and disturbance by air movement. The dishes were held at ambient outdoor air temperature under shaded natural light in a covered area with good air circulation. Experiments were conducted at the normal salinity of Hawaiian waters of from 35‰ to 36‰. Air temperature and water temperature are very close to each other in Kāne‘ohe Bay (Bathen, 1968), thus the experiment simulated normal reef temperatures. The temperature during this experiment held between 25 °C to 27 °C. Larvae prefer low light conditions for settlement (Baker, 1995) so the experiment was conducted under a shed roof with irradiance of approximately 5% of full sunlight as measured with an integrating quanta meter (LI-COR INC.). Thus conditions of temperature, irradiance and salinity were similar in all cases.

At the end of a two week incubation period the surviving planulae larvae and coral recruits (Fig. 3B) were counted. After removing the larvae, the sediment from each Petri dish was washed onto a pre-weighed Whatman GF/F 47 mm glass microfiber filter. The initial dry weight of the filter was subtracted from the weight of the dried filter with sediment and sediment weight normalized to the bottom area of the Petri dish. Larvae percentage survival (total swimming plus total live recruits) and percentage of larvae that settled were calculated. A few of the planulae settled and began to calcify on the surface film of the water as previously reported by Richmond (1985) who attributed this behavior to lack of suitable settlement substrate. These coral recruits were not included in the total coral recruits on substrate, but were included in the survivor total. Only planulae that had settled and calcified on the Petri dish bottom or on the sides were used in the analysis of recruitment versus sediment.

The first five runs were made with 11 different sediment levels. During the last two runs the gradient was reduced to seven different levels. Statistical analysis of the coral settlement data was performed using Minitab™ Version 14 statistical software. The percent settlement and percent survival data were transformed using the arcsine square root transformation of proportional data in order to meet the requirement for normal distribution in the settlement and survival data.

## Results and Discussion

**Figure 4:**
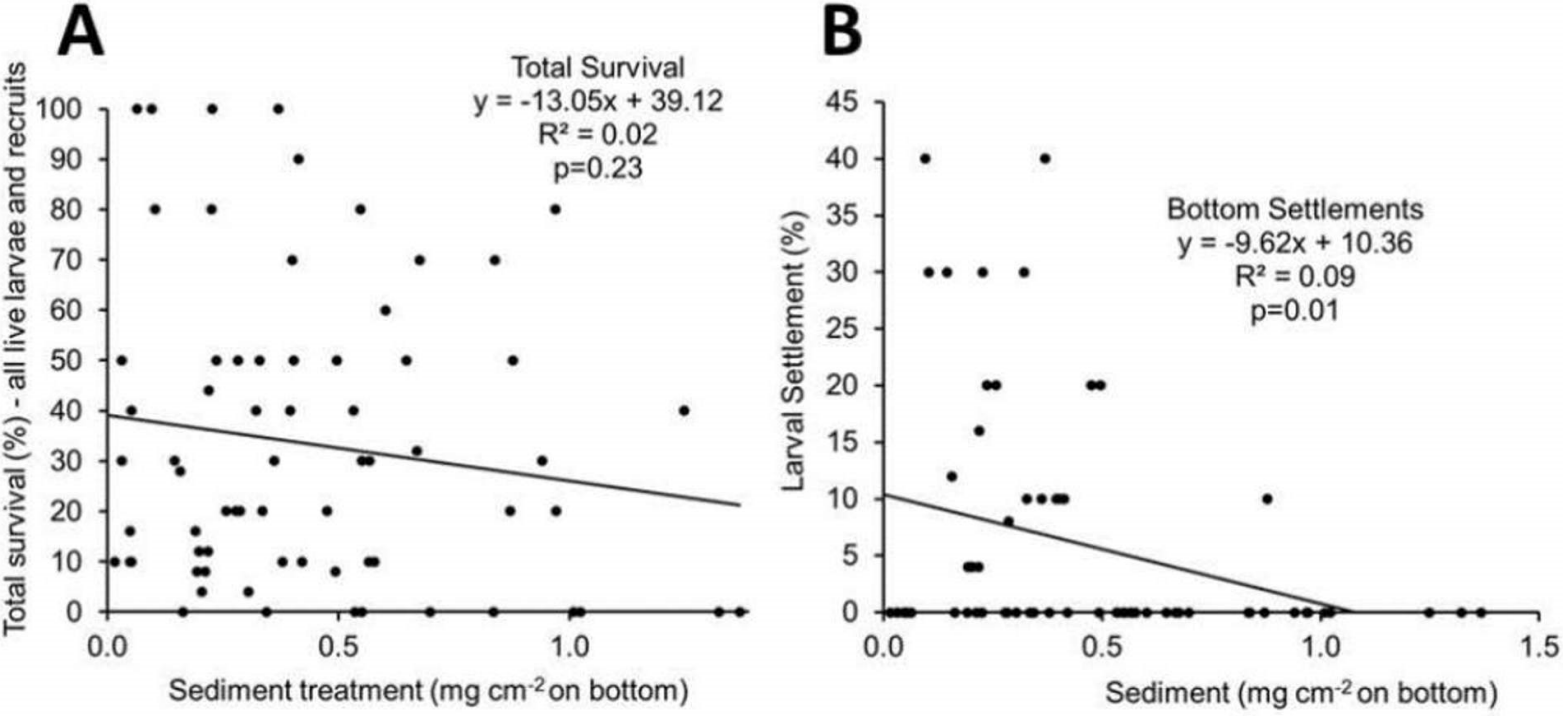
(A) Total larval survival as a function of sediment load (% live larvae and live coral recruits) at the end of each two week incubation. (B) Larval bottom settlement as a function of sediment load (%) at the end of each two week incubation.

Percent survival expressed as sum of live coral recruits and live larvae at the end of each two week experiment (Fig. 4A) did not show significant mortality. Figure 4B shows the relationship between the amounts of sediment covering the bottom of the dish in mg cm^−2^ versus percent of larvae that settled on the bottom. The linear regression analysis showed a significant relationship between the amount of sediment and the percent settlement of larvae at *a* = 0.5, however only 9% of the variability was explained due to the large number of recruitment runs with no settlement (Fig. 4B). No recruitment occurred over sediment films that exceeded 0.9 mg cm^−2^ (Fig. 4B).

**Figure 5:**
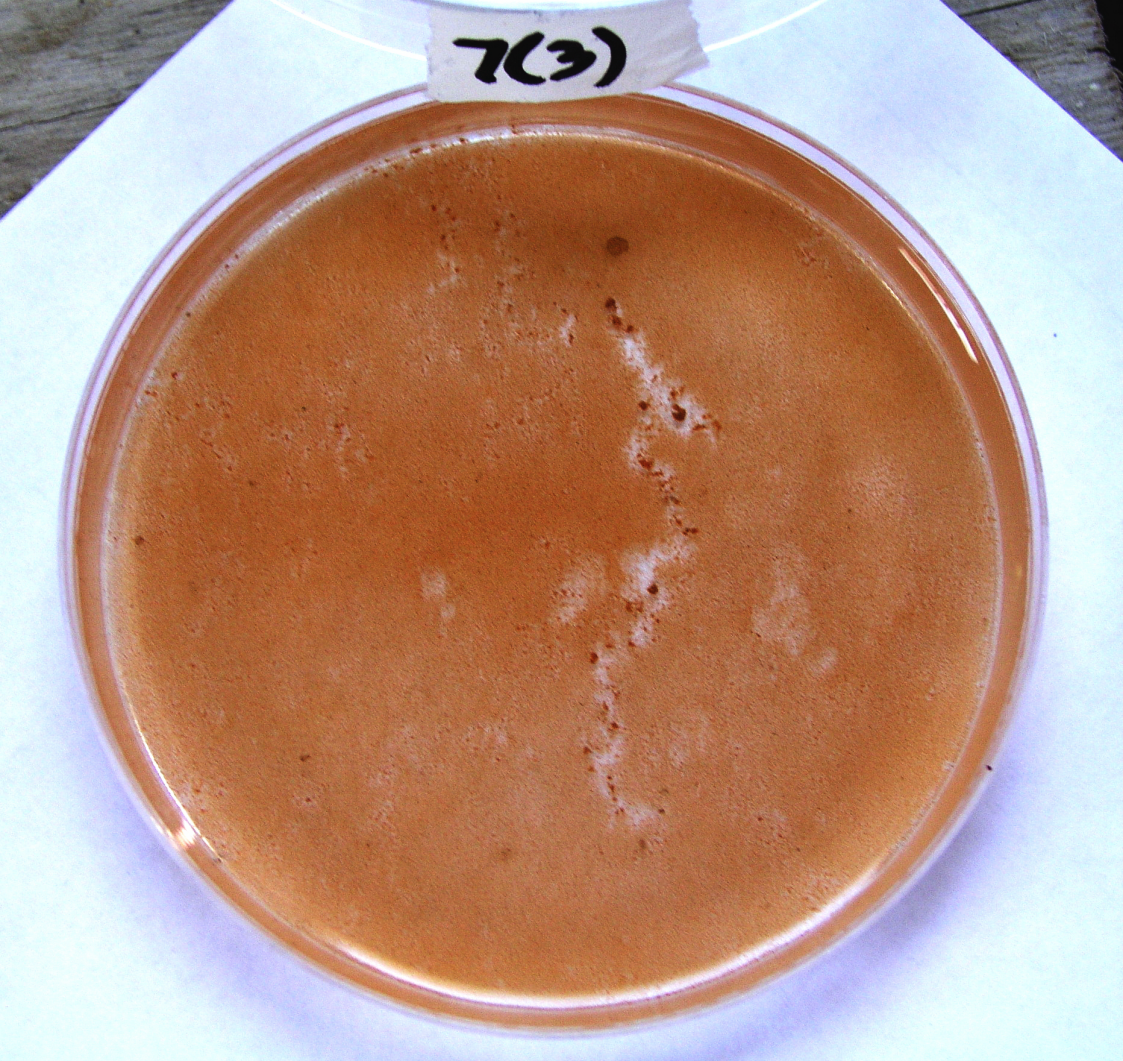
Tracks in the sediment film made by “crawling” planulae larvae. Diameter of Petri dish is 10 cm.

Some of the larvae settled on the sediment-free walls of the Petri dishes, but there was no significant difference between settlements and sediment treatment. We observed that “tracks” were formed on the sediment film in a number of the dishes (Fig. 5) by larvae seeking suitable substrata at both high and low levels of sediment. Planulae are positively attracted to substrate (Edmondson, 1946; Harrigan, 1972). The planulae move about using cilia to seek out a suitable place to settle based on physical and chemical cues. Where sediment covered the biofilm surface, some planulae apparently resorted to “crawling” along the floor of the Petri dish in search of an exposed area, pushing the fine sediment out of their way. In a few instances larvae subsequently settled on these cleared tracks under conditions that normally would block recruitment.

It is well established that “normal” suspended sediment rates on coral reefs range from 10 mg cm^−2^/day or less (Rogers, 1990). At the upper end of this range, the accumulation of sediment on 1 cm^−2^ of surface would be 10 mg unless it is removed by water motion or other disturbances. However, coral recruitment is blocked on surfaces with only 1 mg cm^−2^ of sediment. Corals have been shown to thrive in experiments with very high concentrations of suspended sediment in the lab and the field (Te, 1992; Te, 2001; Hodgson, 1991). In natural environments corals do exist in areas of extremely high sediment (DeMartini et al., 2012; Larcombe, Costen & Woolfe, 2001). Thus, surface sediment seems to be a more critical factor than suspended sediment rates for coral settlement. Other evidence to support this is a unique strategy some corals possess. Under conditions of environmental stress including sedimentation, *P. damicornis* larvae exhibit reverse metamorphosis, a survival mechanism known as “polyp bailout”, where they will release from their calices to resettle onto more suitable substrate. Thus it is possible for this species to settle in highly sedimented environments and still survive where other species may perish.

Sediment resuspension and water motion are critical factors that influence benthic sediment layers. Larcombe et al. (1995) describes the most important factors that control the settlement of suspended sediments as winds and waves. Tidal currents were also found to be important in preventing long-term buildup of sediments. These influences may increase or remove sediment concentrations under which coral larval settlement occurs. Our study shows that when no other factors are involved <0.9 mg cm^−2^ of sediment (0.047 mm thick) will block recruitment completely. Sediments exhibit high spatial and temporal variability due to inconsistency in timing and delivery of sediment sources (stream discharge, land-based run-off, landslides, etc.) and events (rainfall, storm surf, etc.) to nearshore reefs (Storlazzi & Jaffe, 2008). Due to these highly variable fluctuations, natural levels of sedimentation are difficult to ascertain, however typical suspended solids concentrations are <10 mg l^−1^. In the main Hawaiian Islands, Coral Reef Assessment and Monitoring data from 91 stations show a wide range of fine sediment (<63 µm) from 0.1% to 63.1% of the bulk sediment collected (Rodgers, 2005).

Harrington et al. (2004) found that settlement rates of the reef-building corals *Acropora tenuis* and *A. millepora* depended on crustose coralline algae (CCA) species and whether the CCA was alive or dead. Tran & Hadfield (2011) found “larvae of the scleractinian coral *Pocillopora damicornis* require a natural cue from surface-biofilm bacteria to select a suitable substratum on which to attach, metamorphose, and grow into a benthic polyp”. Davies et al. (2014) examined settlement cues for several coral species originating from different ocean provinces and detected significant differences in cue preferences among coral species, even for corals originating from the same reef. The thin layer of fine sediment that blocked settlement of *Pocillopora damicornis* larvae in this experiment raises the possibility that fine sediment films could block cues and impact coral settlement across a wide range of species.

Our laboratory results should be used with caution in applying the results to field situations. However, the value of this research is we show that an extremely thin film can block recruitment.

In summary, the results of the present investigation support and amplify the findings of previous investigations:

• Although a variety of settling cues have been shown it is possible that a thin film of sediment can override these cues blocking larval settlement.
• Recruitment rate of larvae in sediment experiments is very low (Fig. 4). Te (1992) recorded 2% to 3% settlement of *Pocillopora damicornis* larvae over a two week period. Babcock & Davies (1991) report a mean of only 15% settlement in a two day experiment using larvae of the coral *Acropora millepora*.
• There is no significant difference in recruitment versus sedimentation on vertical walls that do not accumulate sediment as shown previously (Te, 1992; Babcock & Davies, 1991).
• Larval behavior is an important factor in determining settling success and survival. Larvae will seek a suitable settlement site (Harrigan, 1972) and will find areas that are free of sediment. Hodgson (1991) conducted larvae settlement experiments on *P. damicornis* on glass plates with sediment layers of 0 to 500 mg cm^−2^, but the sediment covering the glass settling plates was not uniform, having some areas clear of sediment. The corals were able to find and settle in open areas not covered with sediment.
• A relatively thin uniform coating of land-derived sediment can prevent coral recruitment. Hodgson (1991) reports a larval settlement threshold for *P. damicornis* of 536 mg cm^−2^ over patchy horizontal surfaces that blocked 95% of coral settlements. Results of the present study refine this cutoff to a lower value of 1 mg cm^−2^, for a uniform sediment film of fine muds.
